# Robotic versus laparoscopic radical nephroureterectomy for upper tract urothelial carcinoma: comparable oncologic outcomes with reduced intravesical recurrence in pathological T3 disease

**DOI:** 10.1007/s11701-026-03455-w

**Published:** 2026-05-29

**Authors:** I-Hsuan Alan Chen, Hao-Lun Luo, Chi-Hsiang Chu, Chia-Cheng Yu, Tzu-Ping Lin, Chih-Yu Yang

**Affiliations:** 1 Division of Transplant Surgery, Department of Surgery, Kaohsiung Veterans General Hospital, Kaohsiung, Taiwan; 2 Institute of Clinical Medicine, School of Medicine, National Yang Ming Chiao Tung University, Taipei, 112304 Taiwan; 3 School of Medicine, College of Medicine, National Sun Yat-sen University, Kaohsiung, Taiwan; 4 Division of Urology, Department of Surgery, Kaohsiung Veterans General Hospital, Kaohsiung, Taiwan; 5 Department of Urology, Kaohsiung Chang Gung Memorial Hospital, Chang Gung University College of Medicine, Kaohsiung, Taiwan; 6 Institute of Statistics, National University of Kaohsiung, Kaohsiung, Taiwan; 7 Division of Nephrology, Department of Medicine, Taipei Veterans General Hospital, Taipei, Taiwan; 8 Center for Intelligent Drug Systems and Smart Bio-devices (IDS2B), National Yang Ming Chiao Tung University, Hsinchu, Taiwan; 9 Stem Cell Research Center, National Yang Ming Chiao Tung University, Taipei, Taiwan; 10 International Medical Service Center, Taipei Veterans General Hospital, Taipei, Taiwan

**Keywords:** Carcinoma, Transitional cell, Ureteral neoplasms, Robotic surgical procedures, Laparoscopy, Competing risks, Intravesical recurrence

## Abstract

Minimally invasive radical nephroureterectomy (RNU) has been increasingly adopted for upper tract urothelial carcinoma (UTUC); however, direct comparisons between laparoscopic and robot-assisted approaches remain limited. We retrospectively analyzed consecutive patients treated with minimally invasive RNU with bladder cuff excision between August 2014 and July 2025. Patients were stratified into laparoscopic (LNU) and robot-assisted (RANU) groups. Perioperative outcomes and oncologic endpoints, including intravesical recurrence (IVR), disease-free survival (DFS) as well as overall survival (OS), were evaluated. Fine–Gray competing risk regression was applied to assess cancer-specific mortality and IVR. A total of 287 patients were enrolled, comprising 152 in the LNU group and 135 in the RANU group. Perioperative outcomes were comparable between groups, with no significant differences in estimated blood loss, theater time, or major complication rates. During follow-up, no remarkable differences were identified in DFS or OS. Competing risk analysis demonstrated that surgical platform was not significantly correlated with cancer-specific mortality or IVR in the overall cohort. In the subgroup of patients with pathological T3 disease, IVR occurred less frequently in the robotic group compared with the laparoscopic group (7.3% vs. 27.3%, *p* = 0.016). In exploratory multivariable analysis, robot-assisted surgery was associated with a decreased likelihood of IVR (sHR 0.275, *p* = 0.041). This finding should be interpreted cautiously in light of the limited sample size and the post-hoc nature of the analysis. Overall, robot-assisted RNU demonstrated perioperative and oncologic outcomes comparable to laparoscopic surgery. The observed difference in IVR in patients with locally advanced disease is hypothesis-generating and warrants further validation in prospective studies.

**Supplementary Information:**

The online version contains supplementary material available at 10.1007/s11701-026-03455-w.

## Introduction

 Upper tract urothelial carcinoma (UTUC) is comparatively uncommon on a global scale; however, in Taiwan it accounts for a disproportionately high percentage of urothelial malignancies, comprising about 30–40% of cases [1]. Radical nephroureterectomy (RNU) combined with bladder cuff excision (BCE) remains the primary treatment for patients with localized or advanced-stage disease. Laparoscopic RNU, first described by Clayman in 1991, heralded the shift toward minimally invasive surgical (MIS) management of UTUC [2]. Sixteen years later, Rose et al. [3] introduced the initial experience with robotic-assisted RNU, further broadening the MIS armamentarium. Since these landmark advancements, laparoscopic and robotic techniques have gained widespread acceptance and have fundamentally transformed the surgical approach to UTUC over the past two decades.

While MIS approaches for UTUC have become more widely adopted, concerns regarding its oncologic safety compared with conventional open surgery persist. In a nationwide multicenter cohort, encompassing 2,430 patients treated between 1988 and 2022, MIS—comprising laparoscopic and robotic RNU—was associated with improved overall survival (OS), cancer-specific survival (CSS), as well as disease-free survival (DFS) after propensity score matching [4]. Notably, these favorable outcomes were observed notwithstanding the higher prevalence of adverse pathological features in the MIS cohort, including carcinoma in situ (CIS), high-grade tumors, along with tumor necrosis. These findings indicate that minimally invasive platforms achieve oncologic control comparable to, and possibly better than, open surgery when meticulous surgical principles—particularly adequate BCE—are maintained. However, while the oncologic equivalence of MIS and open RNU has been increasingly supported, less is known about potential differences between laparoscopic and robotic platforms, particularly with respect to long-term cancer-specific outcomes and evolving surgical experience over time.

Several contemporary systematic reviews and meta-analyses have attempted to clarify the comparative performance of laparoscopic RNU (LNU) and robot-assisted RNU (RANU); however, the available evidence remains limited by study design and heterogeneity. In a 2023 systematic review including 10 retrospective cohort studies and 29,987 patients, no remarkable differences were found between LNU and RANU with regard to positive surgical margins (9.6% vs. 9.0%), major morbidity (8.4% vs. 8.3%), or overall complications, while perioperative mortality was reported to be lower in the robotic cohort (1.1% vs. 1.8%), although sensitivity analyses attenuated this finding [5]. More recently, a 2025 meta-analysis incorporating 21 studies and 32,882 patients similarly demonstrated comparable operative duration and positive margin rates, with RANU associated with shorter hospital stay (mean difference approximately 1.1 days) and an increased likelihood of undergoing lymph node dissection [6]. Importantly, all included studies were observational in nature, and no randomized controlled trials have directly compared LNU and RANU. Variability in bladder cuff management, extent of lymphadenectomy, and follow-up duration across institutions further complicates interpretation of pooled results.

Beyond the absence of randomized evidence, several methodological limitations of prior studies warrant consideration. Many comparative analyses have been derived from large multicenter registries or administrative databases, where granular pathological characteristics, technical nuances of bladder cuff excision, and surgeon experience are not uniformly captured. Even in studies employing propensity score matching, residual confounding related to tumor stage distribution, lymph node dissection practices, and institutional learning curves cannot be fully excluded. Moreover, long-term oncologic endpoints—such as CSS and recurrence patterns—have been inconsistently reported in previous meta-analyses, and competing risk methodologies have rarely been applied despite the advanced age and substantial comorbidity burden of the UTUC population. In this context, well-characterized single-center cohorts with standardized operative techniques, consistent bladder cuff management, and adequate follow-up remain important to better delineate potential differences between laparoscopic and robotic RNU with BCE.

Therefore, we conducted a single-center comparative study of laparoscopic and robot-assisted RNU with BCE to investigate perioperative and oncologic outcomes between the two MIS platforms. Competing risk modeling was applied to better estimate cancer-specific outcomes while accounting for non–cancer-related mortality. This analysis aimed to provide more methodologically rigorous evidence evaluating the clinical effectiveness of laparoscopic and robotic approaches under standardized surgical conditions.

## Materials and methods

### Study design and patient enrollment

Of the 331 patients evaluated, 287 were enrolled in the final analysis, with 152 receiving LNU and 135 undergoing RANU. All patients included in this study underwent RNU with BCE, and cases treated with segmental ureterectomy and ureteral reimplantation were not included. Clinical and pathological variables, along with perioperative data and oncologic follow-up information, were retrieved from the institutional electronic medical record system. Patients with pathological T4 disease (*n* = 5), lymph node–positive disease (*n* = 19), distant metastasis (*n* = 2), those who had received neoadjuvant therapy (*n* = 17), and patients with bilateral UTUC (*n* = 1) were excluded from the analysis. Approval for this study was obtained from the institutional review board (IRB No.: VGHKS14-CT3-06).

## Surgical technique

### Laparoscopic radical nephroureterectomy

All procedures were undertaken with patients in a modified flank position. A transperitoneal approach was generally adopted. Port placement followed a conventional laparoscopic nephrectomy configuration. The white line of Toldt was dissected first to mobilize the colon medially; following incising Gerota’s fascia, the ureter was identified early in the operation and clipped caudally to lower the risk of tumor spillage into the urinary bladder. The kidney was mobilized along the plane of Gerota’s fascia. The renal vessels were individually dissected and secured using clips or vascular staplers. The ureter was then traced distally toward its crossing over the iliac vessels. Distal ureterectomy with BCE was most commonly performed through an open Gibson incision, via an extravesical approach. Bladder closure was performed using absorbable sutures, ensuring watertight integrity. Additionally, in patients with anticipated dense intraperitoneal adhesions due to prior abdominal surgery, a retroperitoneal approach was selected. In such cases, the Zuckerkandl fascia was identified and opened without prior colonic mobilization. The renal artery was typically identified first, followed by careful dissection and ligation of the renal pedicle. The remaining surgical steps were similar to those of the transperitoneal approach.

### Robot-assisted radical nephroureterectomy

For RANU, patients were placed in a modified flank position to optimize exposure of the upper urinary tract. A 10- or 12-mm assistant port was introduced at the periumbilical region, followed by placement of 4 robotic trocars along the midclavicular line at 6–8 cm intervals under direct vision. An additional assistant port was introduced at the midline between the two upper robotic ports to facilitate suction, retraction, and specimen handling. The robotic cart was docked over the ipsilateral flank, and a 30-degree robotic endoscope was utilized throughout the procedure to provide enhanced depth perception and stable three-dimensional visualization.

Following medial mobilization of the colon, Gerota’s fascia was opened to expose the upper urinary tract. The ureter was located early and controlled distal to the tumor with endoclips to lessen the risk of tumor cell dissemination. The renal hilum was subsequently dissected, and the renal vessels were individually isolated and transected using robotic instruments and vascular stapling devices. Dissection was subsequently continued caudally along the ureter toward the ureterovesical junction. Extravesical bladder cuff excision was performed with meticulous intramural ureteral dissection, followed by watertight bladder closure using running 2 − 0 barbed sutures with intraoperative leak testing to confirm bladder integrity. The specimen was retrieved using an endoscopic bag and removed via an extended port-site incision.

### Data collection and outcome measures

Baseline demographic and clinicopathological data were recorded, including age, sex, tumor features, pathological stage, CIS, lymphovascular invasion (LVI), and surgical margin status. Perioperative parameters comprised estimated blood loss (EBL), theater time, and postoperative complications. The severity of complications was assessed utilizing the Clavien–Dindo grading system, with grade ≥ III regarded as major. Oncologic outcomes included intravesical recurrence-free survival (IVRFS), DFS, and OS. IVRFS was defined as the interval from surgery to the first documented bladder recurrence. DFS was defined as the time from surgery to disease recurrence (excluding IVR), distant metastasis, or death. OS was defined as the time from surgery to death from any cause. Given the advanced age of the UTUC population and the presence of competing non-cancer mortality, competing risk analysis was applied to evaluate CSS, treating death from other causes as a competing event. In addition, subgroup analyses were performed in patients with pathological T3 disease to further assess differences in perioperative and oncologic outcomes between the two surgical approaches.

### Statistical analysis

Continuous data are presented as mean ± standard deviation or median with interquartile range, depending on distribution. Group comparisons were performed utilizing Student’s t-test or the Mann–Whitney U test as applicable. Categorical variables are reported as numbers and percentages and were compared utilizing the chi-square test or Fisher’s exact test when required. Time-to-event outcomes were measured from the date of surgery to the occurrence of the event of interest or the last available follow-up. Survival endpoints, including DFS and OS, were analyzed utilizing the Kaplan–Meier method, with differences assessed by the log-rank test.

For cancer-specific mortality, cumulative incidence functions were constructed with non–UTUC-related death treated as a competing event, and comparisons were performed using Gray’s test. Fine–Gray competing risk models were utilized to assess variables associated with CSS as well as IVR, treating death without IVR as a competing event. Factors with p-values < 0.10 in univariable analyses were enrolled into multivariable models, together with the surgical approach based on clinical relevance. Findings are reported as subdistribution hazard ratios (sHRs) along with 95% confidence intervals (CIs). All analyses were two-sided, with *p* < 0.05 indicating statistical significance. Data analysis was carried out using R software (version 4.5.2; R Foundation for Statistical Computing, Vienna, Austria).

## Results

### Patient demographics and clinical features

A total of 287 patients were enrolled in the final analysis, with 152 (53.0%) undergoing LNU and 135 (47.0%) undergoing RANU. Baseline demographic and clinicopathologic characteristics were broadly similar between both groups (Table 1). The mean age at surgery was 70.6 ± 8.9 years in the laparoscopic cohort and 71.4 ± 10.0 years in the robotic cohort (*p* = 0.303), with a similar proportion of male patients (41.4% vs. 41.5%, *p* > 0.999). Tumor laterality and anatomical distribution were comparable, including renal pelvis, ureter, and combined locations. Ureteral tumor location (proximal, middle, distal) was also evenly distributed. The prevalence of prior bladder cancer history, intravesical chemotherapy instillation, and adjuvant systemic therapy differed between groups, with a higher rate of intravesical chemotherapy observed in the robotic cohort (87.4% vs. 51.3%, *p* < 0.001). Tumor size, grade, and multifocality were similar between groups. Although the distribution of pathological T stage did not reach statistical significance (*p* = 0.160), a higher proportion of T3 disease was noted in the robotic group (40.7% vs. 28.9%). Rates of CIS, LVI, and positive surgical margins were comparable. Overall, no clinically meaningful baseline imbalances were identified.

**Table 1:** Baseline demographic and clinicopathological characteristics

Variable	Laparoscopy	Robot assisted	*P* value
No. patients	152 (53.0%)	135 (47.0%)	
Age	70.6 (8.9)	71.4 (10.0)	0.303
Gender (Male)	63 (41.4%)	56 (41.5%)	> 0.999
Laterality			0.922
Left	74 (48.7%)	64 (47.4%)	
Right	78 (51.3%)	71 (52.6%)	
Tumor location			0.867
Renal pelvis	71 (46.7%)	67 (49.6%)	
Ureter	62 (40.8%)	53 (39.3%)	
Renal pelvis and Ureter	19 (12.5%)	15 (11.1%)	
Location of ureteral tumor			
Proximal	41 (27.0%)	32 (23.7%)	0.618
Middle	28 (18.4%)	24 (17.8%)	> 0.999
Distal	25 (16.4%)	22 (16.3%)	> 0.999
Prior history of bladder cancer	30 (19.7)	20 (14.8)	0.347
Intraoperative intravesical chemotherapy	78 (51.3%)	118 (87.4%)	< 0.001
Adjuvant systemic therapy	30 (19.7%)	31 (23.0%)	0.601
Multifocality	21 (13.0%)	20 (14.0%)	0.943
Tumor size			0.437
< 1 cm	14 (9.2%)	6 (4.4%)	
≥ 1 & < 2 cm	30 (19.7%)	26 (19.3%)	
≥ 2 & < 3 cm	38 (25.0%)	34 (25.2%)	
≥ 3 cm	70 (46.1%)	69 (51.1%)	
High Grade urothelial carcinoma	135 (88.8%)	125 (92.6%)	0.373
T stage			0.160
T0 and CIS	3 (2.0%)	1 (0.7%)	
Ta and T1	75 (49.3%)	59 (43.7%)	
T2	30 (19.7%)	20 (14.8%)	
T3	44 (28.9%)	55 (40.7%)	
Carcinoma in situ	64 (42.1%)	67 (49.6%)	0.247
Lymphovascular invasion	20 (13.2%)	26 (19.3%)	0.213
Positive surgical margin	5 (3.3%)	3 (2.2%)	0.727

### Perioperative outcomes

Perioperative outcomes are summarized in Table 2. Estimated blood loss was numerically lower in the robotic group (110.7 ± 108.5 mL) compared with the laparoscopic group (150.3 ± 180.8 mL), although no statistically significant difference was observed (*p* = 0.188). Theater time was comparable between the two approaches (366.4 ± 91.6 vs. 385.2 ± 107.9 min, *p* = 0.109). Postoperative complications occurred at similar rates (52.0% vs. 53.3%, *p* = 0.911). The distribution of complication severity, assessed using the Clavien–Dindo classification, was comparable at both 30 and 90 days. Most complications were low grade (Clavien–Dindo I–II), while higher-grade complications (≥ III) were infrequent. One Grade V complication occurred in the laparoscopic group.

**Table 2:** Perioperative and oncologic outcomes

Variable	Laparoscopy	Robot assisted	*P* value
Estimated blood loss	150.3 (180.8)	110.7 (108.5)	0.188
Theater time (min)	366.4 (91.6)	385.2 (107.9)	0.109
Complication	79 (52.0%)	72 (53.3%)	0.911
Max. grade of Complication (30 days)			0.993
No	73 (48.0%)	63 (46.7%)	
Grade 1	37 (24.3%)	36 (26.7%)	
Grade 2	36 (23.7%)	32 (23.7%)	
Grade 3	3 (2.0%)	2 (1.5%)	
Grade 4	3 (2.0%)	2 (1.5%)	
Max. grade of Complication (90 days)			0.770
No	71 (46.7%)	60 (44.4%)	
Grade 1	35 (23.0%)	38 (28.1%)	
Grade 2	37 (24.3%)	33 (24.4%)	
Grade 3	4 (2.6%)	2 (1.5%)	
Grade 4	3 (2.0%)	2 (1.5%)	
Grade 5	2 (1.3%)	0 (0.0%)	
Intravesical recurrence	38 (25.0%)	24 (17.8%)	0.180
Disease recurrence	14 (9.2%)	9 (6.7%)	0.566
Metastasis	20 (13.2%)	23 (17.0%)	0.451
Lymph node	12 (7.9%)	12 (8.9%)	0.928
Bone	7 (4.6%)	7 (5.2%)	> 0.999
Liver	3 (2.0%)	3 (2.2%)	> 0.999
Lung	3 (2.0%)	8 (5.9%)	0.152
Others	5 (3.3%)	4 (3.0%)	> 0.999
Mortality			0.896
UTUC specific	10 (6.6%)	10 (7.4%)	
Other causes	12 (7.9%)	9 (6.7%)	
Follow-up (years)	4.4 (2.8)	3.8 (2.7)	0.061

Max, maximum; UTUC, upper tract urothelial carcinoma

### Oncologic outcomes

**Fig. 1:**
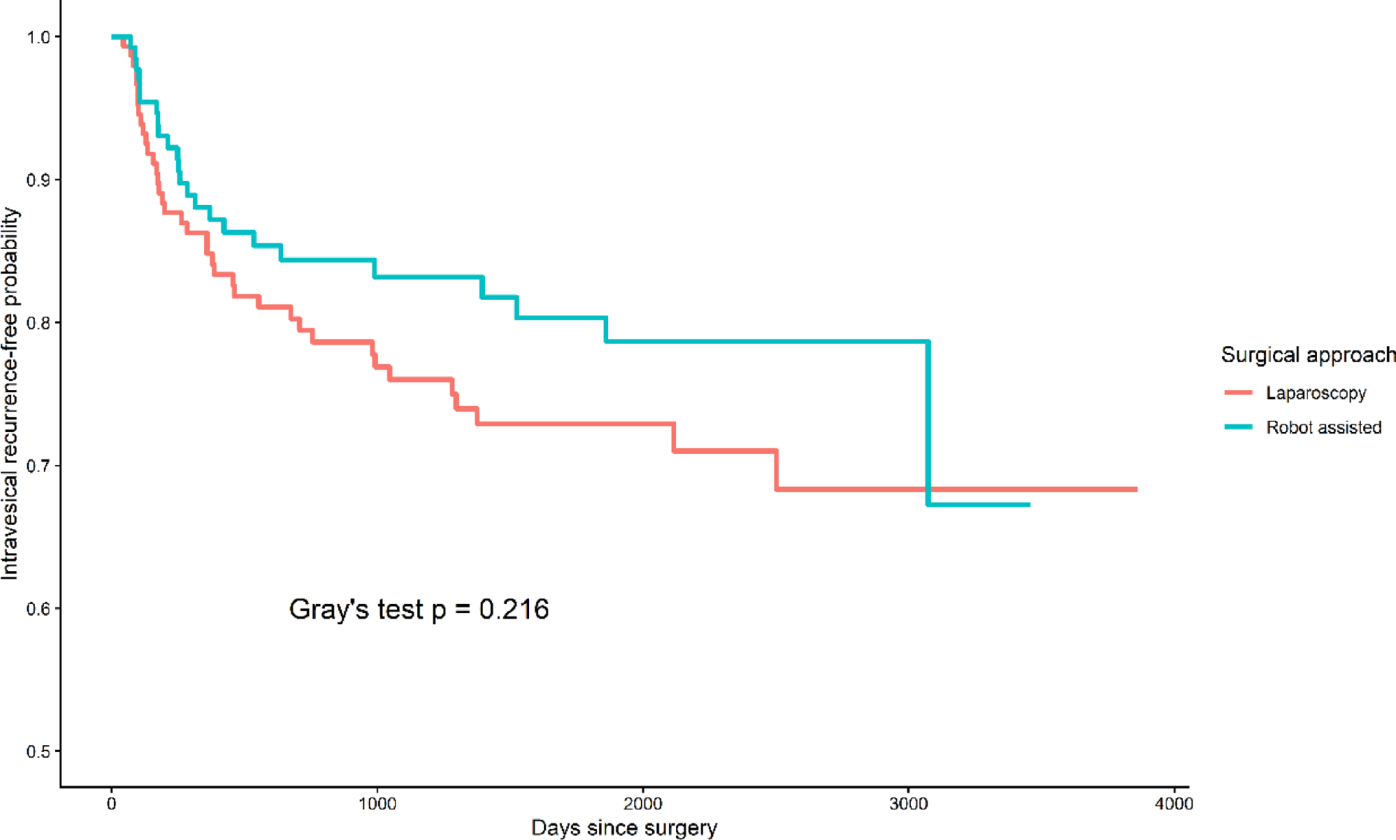
Cumulative incidence of intravesical recurrence accounting for competing risks

A longer duration of follow-up was observed in the LNU group relative to the RANU group (median 4.4 vs. 3.8 years, *p* = 0.061). Intravesical recurrence (IVR) occurred in 25.0% of patients undergoing laparoscopic surgery and 17.8% of those undergoing robotic surgery, representing a non-significant trend toward lower recurrence in the robotic cohort (*p* = 0.180). When accounting for competing risks, cumulative incidence analysis similarly revealed no significant difference in IVR between both groups (Fig. 1). Rates of non-bladder recurrence and distant metastasis were comparable. UTUC recurrence outside the bladder occurred in 9.2% vs. 6.7% of patients (*p* = 0.566), and distant metastasis occurred in 13.2% vs. 17.0% (*p* = 0.451). Patterns of metastatic spread were similar between groups. Cancer-specific mortality was observed in 6.6% of patients in the laparoscopic group and 7.4% in the robotic group, with similar rates of non–cancer-related mortality. No differences in DFS or OS were identified between the two approaches using Kaplan–Meier analysis.

### Competing risk analysis for cancer-specific mortality

**Fig. 2:**
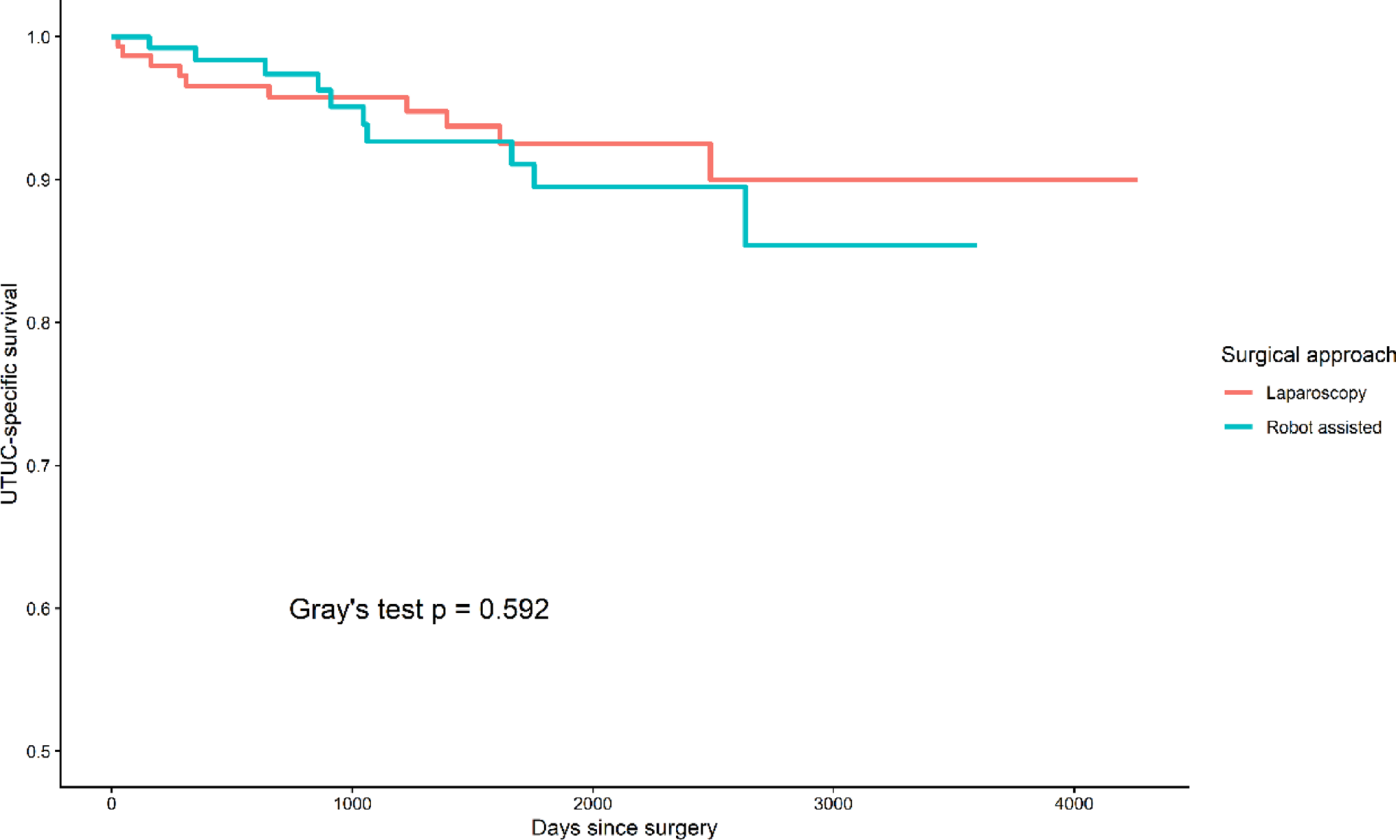
Cumulative incidence of cancer-specific mortality accounting for competing risks

To address the potential impact of non–cancer-related mortality as a competing event, Fine–Gray regression was performed (Supplementary Table S3). The type of surgical approach was not significantly associated with cancer-specific mortality in either univariable or multivariable models. In univariable analysis, pathological T3 disease was linked to a higher risk of cancer-specific death, while LVI showed borderline significance; however, this effect was not maintained after adjustment. Consistent with these findings, cumulative incidence analysis did not demonstrate a significant difference in cancer-specific mortality between the two groups (Fig. 2).

### Subgroup analysis of pathological T3 disease

**Fig. 3:**
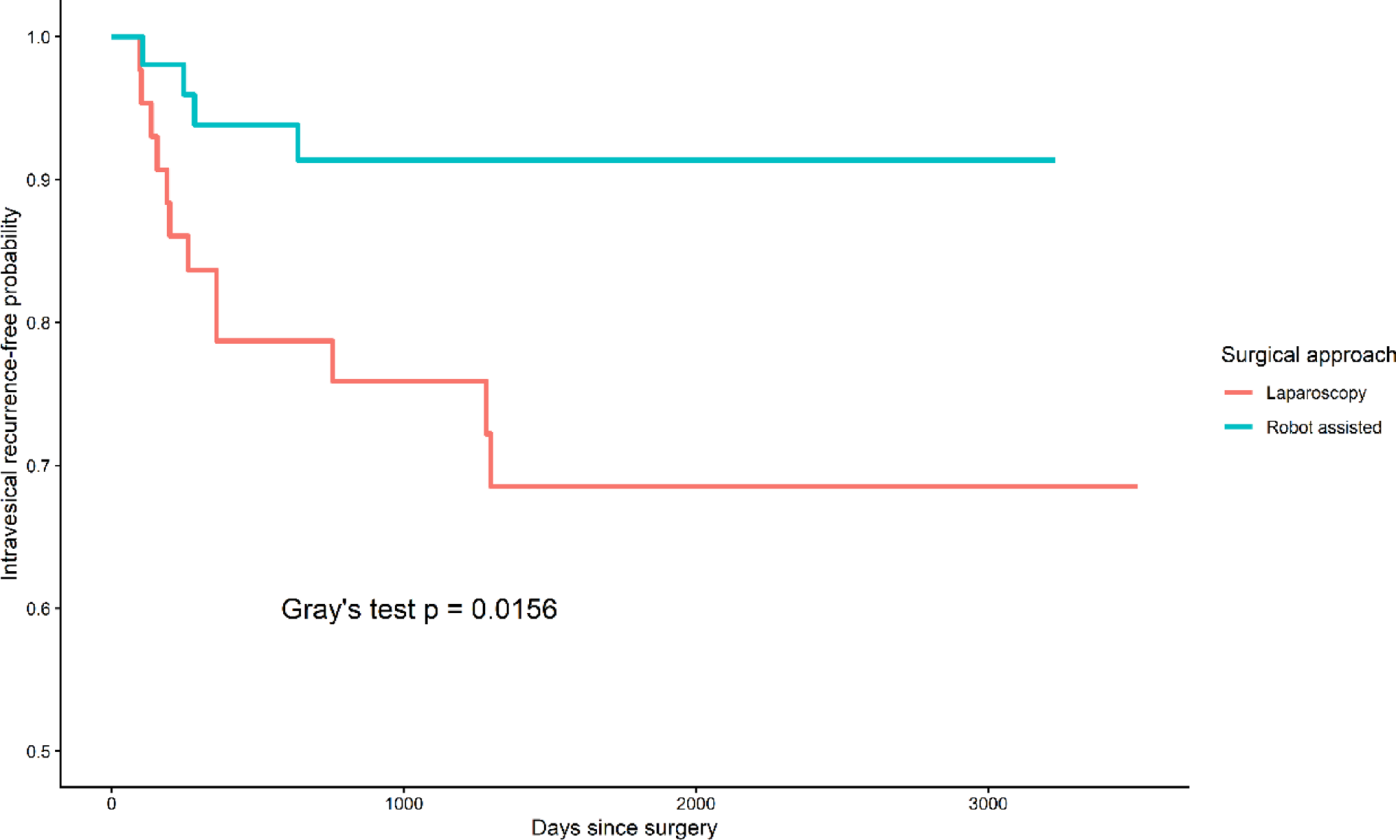
Cumulative incidence of intravesical recurrence in patients with pathological T3 disease, accounting for competing risks

In light of the higher proportion of pathological T3 disease in the robotic cohort, a dedicated subgroup analysis was conducted. Within this subset, baseline characteristics and perioperative outcomes remained comparable between the two groups (Supplementary Tables S1 and S2). Notably, intravesical recurrence (IVR) demonstrated a marked difference, with a recurrence rate of 27.3% in the laparoscopic group compared with 7.3% in the robotic group (*p* = 0.016). Competing risk analysis further confirmed a significantly lower cumulative incidence of IVR in patients undergoing robotic surgery (Fig. 3), whereas rates of systemic recurrence and mortality were similar between the two approaches.

To further explore factors associated with IVR in this subgroup, Fine–Gray competing risk regression was performed, treating death without IVR as a competing event (Table 3). In univariable analysis, robot-assisted surgery was correlated with a lower risk of IVR (sHR 0.269, 95% CI 0.088–0.820, *p* = 0.021). In contrast, male sex (sHR 6.486, *p* = 0.001), prior history of bladder cancer (sHR 3.256, *p* = 0.028), and carcinoma in situ (sHR 5.865, *p* = 0.023) were related to an increased risk of recurrence. In multivariable analysis, robot-assisted surgery remained associated with a reduced risk of IVR (sHR 0.275, 95% CI 0.080–0.951, *p* = 0.041), while male sex remained an independent predictor of higher recurrence risk (sHR 5.526, *p* = 0.002). Carcinoma in situ and prior bladder cancer history showed borderline significance after adjustment.

**Table 3:** Fine–Gray competing risk regression analysis for IVR in patients with pathological T3 disease

Variable	Univariable sHR	95% CI	*P* value	Multivariable sHR	95% CI	*P* value
Robotic surgical platform	0.269	0.088–0.820	0.021	0.275	0.080–0.951	0.041
Age	1.025	0.975–1.077	0.340	—	—	—
Gender (Male)	6.486	2.165–19.437	0.001	5.526	1.869–16.341	0.002
Distal ureteral tumor	0.697	0.167–2.917	0.620	—	—	—
Prior history of BCa	3.256	1.137–9.320	0.028	3.503	0.805–15.234	0.095
Intra-op intravesical CT	0.523	0.197–1.387	0.190	2.179	0.521–9.109	0.290
Adjuvant systemic therapy	0.709	0.268–1.872	0.490	—	—	—
Tumor size (ref: <2 cm)						
≥ 2 & <3 cm	5.161	0.604–44.078	0.130	—	—	—
≥ 3 cm	4.462	0.598–33.290	0.140	—	—	—
Carcinoma in situ	5.865	1.280–26.878	0.023	5.035	0.875–28.971	0.070
Lymphovascular invasion	0.504	0.163–1.561	0.240	—	—	—

BCa, bladder cancer; CT, chemotherapy; CI, confidence interval; Intra-op, intraoperative.; sHR, subdistribution hazard ratio

## Discussion

In this single-center cohort, we compared perioperative and oncologic outcomes between laparoscopic and robotic nephroureterectomy for UTUC. Overall, both approaches demonstrated comparable safety profiles and similar long-term oncologic outcomes across multiple endpoints. While no significant differences were reported in DFS, OS, or cancer-specific mortality, a lower incidence of IVR was noted in patients with pathological T3 disease undergoing robotic surgery. Given the nature of the analysis, this finding should be interpreted cautiously and considered exploratory.

### Perioperative safety and comparative outcomes

Our perioperative outcomes are consistent with previous reports supporting minimally invasive nephroureterectomy as a safe surgical approach [7]. EBL, theater time, and complication rates were similar between both groups, and major complications remained uncommon. These findings align with prior meta-analyses suggesting that both laparoscopic and robotic approaches provide acceptable perioperative safety, with only modest differences in selected parameters such as blood loss or hospital stay [5, 6, 8]. Taken together, our results further support the role of robotic surgery as a feasible minimally invasive alternative rather than a clearly superior modality in terms of perioperative outcomes. From an oncologic perspective, we did not observe significant differences between the two approaches in survival outcomes. This is in line with existing literature [9] indicating that long-term oncologic results are largely comparable between laparoscopic and robotic nephroureterectomy when appropriate oncologic principles are followed.

### Methodological refinements: competing risk analysis

Another important aspect of the present study is the utilization of competing risk analysis to evaluate cancer-specific mortality as well as IVR. Patients with UTUC are often elderly and frequently harbor multiple comorbidities, resulting in a substantial risk of non–cancer-related mortality. Conventional Kaplan–Meier survival analyses may therefore overestimate these oncologic outcomes. By applying Fine–Gray competing risk regression, we aimed to provide a more accurate assessment of cancer-specific mortality and IVR. In this analysis, surgical approach was not significantly related to these outcomes in overall population, whereas tumor-related factors, such as pathological stage, demonstrated stronger associations in univariable analyses. Overall, these results reinforce that tumor biology, rather than the surgical platform, remains the dominant determinant of prognosis.

### Intravesical recurrence and the T3 subgroup

With respect to IVR, we observed a lower rate of IVR in the robotic group within the pathological T3 subgroup. IVR remains a common event following nephroureterectomy, with reported rates ranging from approximately 22%–47% as reported in the majority of studies [10, 11]. Although several mechanisms such as tumor cell implantation, field cancerization, and intraluminal seeding have been proposed, these processes are difficult to isolate in retrospective analyses. In the present study, ureteral tumor location and baseline clinicopathologic characteristics were comparable between groups, making it less likely that these factors alone explain the observed difference. One possible explanation relates to technical aspects of distal ureter management and BCE. Although an extravesical approach was consistently used, subtle differences in surgical execution cannot be excluded. Robotic systems offer enhanced visualization and instrument articulation, which may facilitate more controlled dissection and suturing. These factors could theoretically reduce tumor spillage or improve closure integrity, particularly in patients with larger tumor burden such as those with T3 disease. However, as these variables were not directly measured, such interpretations remain speculative [12].

A review of distal ureter management techniques has emphasized the importance of meticulous intramural dissection and early ureteral control in minimizing tumor dissemination. The technical characteristics of robotic surgery may facilitate adherence to these principles, although direct evidence remains limited. Prior institutional and multicenter studies have suggested that RANU may be associated with lower IVR rates, but findings have been inconsistent across cohorts [13, 14]. In addition, studies focusing on locally advanced disease have shown that minimally invasive approaches can achieve oncologic outcomes comparable to open surgery in patients with T3 or T4 tumors [15]. In this context, our findings add to the existing literature but should be interpreted with caution, particularly given the retrospective design and subgroup nature of the analysis. Maida et al. demonstrated favorable outcomes of robotic management for locally advanced UTUC in European high-volume centers [12]. Given the relatively uniform surgical practice and detailed clinicopathologic data at our institution, this single-center cohort may reduce some of the variability observed in multicenter analyses. The use of competing risk models further allowed for a more clinically relevant assessment of cancer-specific mortality and IVR in an elderly population with substantial competing risks. In addition, the focused subgroup analysis in patients with pathological T3 disease provides additional insight into potential differences between surgical approaches in higher-risk settings. Nevertheless, these findings should be regarded as exploratory and hypothesis-generating.

### Perioperative intravesical and systemic therapy

One pivotal consideration is the use of perioperative intravesical chemotherapy. In our cohort, intravesical instillation was not uniformly applied and was more frequently administered in the robotic group. This reflects evolving clinical practice patterns over time rather than a protocol-driven strategy. Although this variable was included in the multivariable model, residual confounding cannot be excluded, and the magnitude of its effect relative to surgical factors remains uncertain. Notably, the direction of effect for intravesical chemotherapy was reversed after adjustment, suggesting the presence of underlying bias and possible model instability given the limited number of events. It is therefore possible that differences in intravesical chemotherapy use may have contributed, at least in part, to the observed variation in IVR. This is particularly relevant in light of randomized data demonstrating a reduction in bladder recurrence with immediate postoperative intravesical chemotherapy [7, 16]. Perioperative systemic therapy represents another potential source of variability in UTUC management [17, 18]. The use of neoadjuvant or adjuvant chemotherapy in our cohort was not standardized and likely evolved over time, influenced by tumor stage, patient condition, and changing treatment practices. While these variables were included when available, differences in treatment timing and selection may still introduce residual confounding. In particular, systemic therapy may affect disease control beyond the primary surgical field, making it difficult to isolate the effect of surgical approach alone. Therefore, perioperative systemic therapy should be considered when interpreting oncologic outcomes, especially in subgroup analyses.

### Limitations

This study has several limitations that should be considered. Given its retrospective design, selection bias and residual confounding cannot be fully excluded. Although baseline characteristics were generally comparable between groups, differences related to case selection, surgeon preference, and evolving clinical practice over time cannot be fully excluded. The robotic approach was introduced later during the study period, resulting in shorter follow-up and potential era-related effects, including changes in perioperative management and learning curve influences. In particular, the higher proportion of T3 disease in the robotic cohort may reflect temporal trends or case selection patterns, which could have contributed to the observed findings in subgroup analyses. In addition, perioperative management, including the use of intravesical chemotherapy, was not standardized and may have varied over time, introducing further heterogeneity. The relatively small number of events, especially within post-hoc subgroup analyses, also limits statistical power and warrants cautious interpretation. Importantly, the observed association between robotic surgery and reduced IVR should not be interpreted as causal, as unmeasured technical and perioperative factors may have influenced the results.

Despite these limitations, this study provides additional real-world evidence supporting the role of minimally invasive nephroureterectomy in UTUC. Both laparoscopic and robotic approaches achieved comparable oncologic outcomes while maintaining acceptable perioperative safety. The lower rate of IVR observed in patients with pathological T3 disease undergoing robotic surgery is of interest; nevertheless, this finding should be regarded as exploratory and hypothesis-generating. Further validation in larger, preferably prospective studies with standardized perioperative management is required before definitive conclusions can be drawn.

### Conclusions

Taken together, robot-assisted RNU demonstrates perioperative safety and long-term oncologic outcomes comparable to those of laparoscopic surgery for UTUC. In the subgroup of patients with pathological T3 disease, a lower incidence of IVR was observed in the robotic cohort; however, this finding should be interpreted with caution given the exploratory nature of the analysis. The observed difference may reflect unmeasured factors related to surgical technique, perioperative management, or patient selection rather than the surgical platform itself. Further prospective studies with larger sample sizes, longer follow-up, and standardized perioperative protocols are warranted to better clarify the potential role of robotic surgery in patients with advanced UTUC.

## Electronic Supplementary Material

Below is the link to the electronic supplementary material.

Supplementary Material 1

## Data Availability

Access to the study data is restricted owing to institutional policies and patient privacy considerations, but the corresponding author may provide the data upon reasonable request and with approval from the IRB.
