# Supplementary material for: Robotic versus laparoscopic radical nephroureterectomy for upper tract urothelial carcinoma: comparable oncologic outcomes with reduced intravesical recurrence in pathological T3 disease
Source: J Robot Surg. 2026 May 29;20(1):549. doi: 10.1007/s11701-026-03455-w (PMC13219172; doi:10.1007/s11701-026-03455-w)
Supplement: Supplementary file 1 — Supplementary Material 1 [file 11701_2026_3455_MOESM1_ESM.docx]

**Supplementary Table S1** Baseline characteristics of patients with pathological T3 disease

| Variable | Laparoscopy | Robot assisted | P value |
| --- | --- | --- | --- |
| No. patients | 44 (44.4%) | 55 (55.6%) |  |
| Age | 71.1 (9.8) | 71.3 (9.7) | 0.880 |
| Gender (Male) | 26 (59.1%) | 36 (65.5%) | 0.659 |
| Laterality |  |  | 0.200 |
| Left | 25 (56.8%) | 23 (41.8%) |  |
| Right | 19 (43.2%) | 32 (58.2%) |  |
| Tumor location |  |  | 0.569 |
| Renal pelvis | 22 (50.0%) | 29 (52.7%) |  |
| Ureter | 13 (29.5%) | 19 (34.5%) |  |
| Renal pelvis and Ureter | 9 (20.5%) | 7 (12.7%) |  |
| Location of ureteral tumor |  |  |  |
| Proximal | 8 (18.2%) | 9 (16.4%) | >0.999 |
| Middle | 6 (13.6%) | 9 (16.4%) | 0.925 |
| Distal | 12 (27.3%) | 12 (21.8%) | 0.694 |
| Prior history of bladder cancer | 8 (18.2%) | 6 (10.9%) | 0.458 |
| Intraoperative intravesical chemotherapy | 24 (54.5%) | 47 (85.5%) | 0.002 |
| Adjuvant systemic therapy | 20 (45.5%) | 26 (47.3%) | >0.999 |
| Multifocality | 11 (25.0%) | 16 (29.1%) | 0.820 |
| Tumor size |  |  | 0.978 |
| <1cm | 1 (2.3%) | 1 (1.8%) |  |
| ≥1 & < 2 cm | 8 (18.2%) | 10 (18.2%) |  |
| ≥2 & < 3 cm | 7 (15.9%) | 11 (20.0%) |  |
| ≥ 3cm | 28 (63.6%) | 33 (60.0%) |  |
| Carcinoma in situ | 25 (56.8%) | 29 (52.7%) | 0.839 |
| Lymphovascular invasion | 16 (36.4%) | 22 (40.0%) | 0.872 |
| Positive surgical margin | 3 (6.8%) | 2 (3.6%) | 0.653 |

**Supplementary Table S2** Perioperative and oncologic outcomes in pathological T3 subgroup

| Variable | Laparoscopy | Robot assisted | P value |
| --- | --- | --- | --- |
| Estimated blood loss | 169.5 (166.3) | 99.7 (80.6) | 0.075 |
| Theater time (min) | 359.4 (100.1) | 384.3 (127.5) | 0.207 |
| Complication | 23 (52.3%) | 23 (41.8%) | 0.405 |
| Max. grade of Complication (30 days) |  |  | 0.485 |
| No | 21 (47.7%) | 32 (58.2%) |  |
| Grade 1 | 10 (22.7%) | 13 (23.6%) |  |
| Grade 2 | 9 (20.5%) | 9 (16.4%) |  |
| Grade 3 | 2 (4.5%) | 0 (0.0%) |  |
| Grade 4 | 2 (4.5%) | 1 (1.8%) |  |
| Max. grade of Complication (90 days) |  |  | 0.340 |
| No | 20 (45.5%) | 32 (58.2%) |  |
| Grade 1 | 9 (20.5%) | 13 (23.6%) |  |
| Grade 2 | 10 (22.7%) | 9 (16.4%) |  |
| Grade 3 | 2 (4.5%) | 0 (0.0%) |  |
| Grade 4 | 2 (4.5%) | 1 (1.8%) |  |
| Grade 5 | 1 (2.3%) | 0 (0.0%) |  |
| Intravesical recurrence | 12 (27.3%) | 4 (7.3%) | 0.016 |
| Disease recurrence | 3 (6.8%) | 4 (7.3%) | >0.999 |
| Metastasis | 10 (22.7%) | 14 (25.5%) | 0.937 |
| Mortality |  |  | 0.937 |
| UTUC specific | 6 (13.6%) | 7 (12.7%) |  |
| Other causes | 4 (9.1%) | 4 (7.3%) |  |
| Follow-up (years) | 4.0 (2.9) | 3.3 (2.5) | 0.250 |

**Supplementary Table S3** Fine–Gray competing risk regression analysis for cancer-specific mortality

| Variable | Univariable sHR | 95% CI | p-value | Multivariable sHR | 95% CI | P value |
| --- | --- | --- | --- | --- | --- | --- |
| Robotic surgical platform | 1.267 | 0.525–3.060 | 0.600 | 1.086 | 0.444–2.661 | 0.860 |
| Age | 0.985 | 0.939–1.034 | 0.550 | — | — | — |
| Gender (Male) | 1.802 | 0.731–4.446 | 0.200 | — | — | — |
| Intra-op intravesical CT | 0.722 | 0.307–1.697 | 0.450 | — | — | — |
| Prior history of BCa | 1.622 | 0.598–4.402 | 0.340 | — | — | — |
| Tumor size (ref: <1 cm) |  |  |  |  |  |  |
| ≥1 & <2 cm | 0.353 | 0.051–2.458 | 0.290 | — | — | — |
| ≥2 & <3 cm | 0.703 | 0.139–3.569 | 0.670 | — | — | — |
| ≥3 cm | 0.930 | 0.208–4.158 | 0.920 | — | — | — |
| T3 stage | 4.086 | 1.662–10.045 | 0.002 | 3.765 | 1.289–10.995 | 0.015 |
| Carcinoma in situ | 1.520 | 0.635–3.638 | 0.350 | — | — | — |
| Lymphovascular invasion | 2.488 | 0.968–6.393 | 0.058 | 1.216 | 0.399–3.710 | 0.730 |

BCa, bladder cancer; CT, chemotherapy; CI, confidence interval; Intra-op, intraoperative.; sHR, subdistribution hazard ratio.
